# Excess length of stay due to methicillin-resistant *Staphylococcus aureus* (MRSA) infection at a large Swiss hospital estimated by multi-state modelling

**DOI:** 10.1186/1753-6561-5-S6-O89

**Published:** 2011-06-29

**Authors:** M Macedo-Vinas, G De Angelis, C Fankhauser, E Safran, J Schrenzel, D Pittet, S Harbarth

**Affiliations:** 1 Hopitaux Universitaires de Geneve, Geneva, Switzerland

## Introduction / objectives

Excess LoS related to MRSA infections increases hospital costs. Current statistical approaches to estimate excess LoS suffer from methodological limitations including time-dependant bias. We aimed to estimate excess LoS due to MRSA infections in acute care wards at the University of Geneva Hospitals using a novel multistate modelling strategy.

## Methods

During 2009, 167 MRSA-infected and 25766 MRSA-uninfected control patients were included in a multistate model where occurrence of MRSA infection was the time-dependent exposure and discharge or death was the study endpoint. Infections were stratified by anatomical site. Excess LoS was extracted computing the Aalen-Johansen estimator of the matrix of transition probabilities. 95% confidence intervals were derived by bootstrap re-sampling. Multivariate Cox regression analysis (adjusted for sex, age, cancer and diseases of the skin and subcutaneous tissue and of circulatory and digestive system) was used to assess the independent effect of MRSA infection on excess LoS.

## Results

Median LoS of infected patients was 30 days compared to 6 days for controls. In the multistate model, excess LoS for all MRSA infections was 11.5 days (95% CI, 7.9-15). The highest impact was due to bacteraemia (20 days; 95% CI 8-32) and skin and soft tissue infections (18.8 days; 95% CI 6.1-31.6). The multivariate Cox proportional hazards model confirmed that nosocomial MRSA infection significantly reduced the likelihood of discharge (adjusted HR 0.69; 95% CI 0.59-0.81).

## Conclusion

Using a novel multistate modelling strategy to avoid time-dependant bias, MRSA infection at any anatomical site proved to significantly prolong LoS in acute care.

## Disclosure of interest

None declared.

